# Homœpathy

**Published:** 1854-05

**Authors:** 


### HOMŒOPATHY.

---

Prof. Henderson, of the University of Edinburgh, is out in reply to his colleague, Prof. Simpson, whose work on Homœopathy we noticed a short time since. The reply is entitled "*Homœopathy fairly represented*," and has been republished by Messrs. Lindsay & Blakiston in a very handsome style. While we admit that the Professor of General Pathology in the University of Edinburgh is quite as able to do justice to Homœopathy as the ablest of its advocates, we must be permitted to say that his "reply" is very little to our taste; and we say this, not, we think, from prejudice against the subject, but because the temper in which it is written is bad, the argument feeble and unfair, and the style verbose and tedious. As to discovering anything new in support of this strange delusion, that was hardly to have been expected even from the learned Edinburgh professor, and we are therefore not disappointed in finding that the main fact insisted upon in his book as favoring homæopathy, is the fact so often urged—the frequent recovery of patients under this system of treatment. This is granted, as we know just as well that patients often recover from disease without any treatment at all; but that Dr. Henderson should contend gravely and laboriously for the efficacy of medicine in infinitesimal doses, we confess surprises us.

And yet we do not know that there is cause for being surprised at *any* vagary of the human mind. Dr. HENDERSON is a physician of learning and note, highly educated and of mature years; but physicians before him, with all his accomplishments and advantages, have been believers in clairvoyance and metallic tractors. It is sad to see such a man so led astray, but we see men, in our times, full of learning and years, becoming dupes to the monstrous absurdities of spiritualism. We may deplore the spectacles, but such things seem to be inseparable from human nature.

Dr. HENDERSON has the usual platitudes about the repugnance of the mind to new truths, the opposition to great discoveries, &c., which are in the mouths of neurologists, mesmerists, and all that class of pretenders. Every humbug offered to the acceptance of men is placed, by its fond author, in the same category with the Copernican system, and HARVEY'S discovery of the circulation of the blood. It must expect its obloquy and buffetings. Truly the opposition to homœopathy continues a great while, and is far from being on the decline for all that we can see.

We must let Dr. HENDERSON speak for himself in one paragraph. It contains an apology for HAHNEMANN—a precious admission that he was not perfect and did not understand everything, and affords a fair specimen of the style and spirit of his work.

"We cease to wonder, then, that even HAHNEMANN, with all his genius and learning, and keen-eyed scientific instinct, *overlooked a few things* in medical science, which he might have studied with advantage to his reputation, and the credit of his system. In the brightness of his own discoveries, these inferior objects "hide their diminished heads," and the eye that dwelt so long and so intently on the light that appeared amidst the general gloom, from its first faint glimmering in its path, as he meditated mournfully on the surrounding chaos where "darkness brooked," on through its gradual kindling into the dawn of a better day to the patient humanity, may well be pardoned its insensibility to the ineffectual glowworm lights that could do little else in his day than make the darkness visible. This plea is specially applicable to his contempt for morbid anatomy, and to his ignoring of ordinary palliations that are capable of producing temporary ease in incurable organic diseases. With him the office of the physician was to *cure;* on that grand consummation his heart was set, and he had no eyes for any end short of the test that could be wished for. Morbid anatomy presupposes death; and whatever light the scalpel shed on disease, to HAHNEMANN it showed only the discomfiture, the imperfection, sometimes even the deadly error of the art that should have healed. He sought only for the means of *curing* diseases, and believed that means existed which, known and rightly used, were equal, by the goodness of the Almighty, ('compared to which,' he says, 'the tenderest mother's love is as thick clouds beside the glory of the noon-day sun,') to the cure of almost all maladies: so that morbid anatomy was in his opinion merely the proof, if not the result of error."

One extract must suffice, and with it we dismiss the book. It will no doubt be a great comfort to the fraternity of homœopathists, but our impression is that few physicians who have other employment will have the patience to wade through its wearisome pages. We venture the prediction that it will not make one convert to Homœopathy.

